# Comparative effectiveness of school- and office-based technology-enhanced interventions for physical activity promotion: A systematic review and meta-analysis

**DOI:** 10.1016/j.pmedr.2026.103409

**Published:** 2026-02-13

**Authors:** Mitch Wyatt, Mickey Bolyard, Lingyi Fu, Hayley Almes, Frank Adutwum, Charles Rodgers, Ryan D. Burns

**Affiliations:** Department of Health and Kinesiology, University of Utah, 1850 East 250 South, Salt Lake City, Utah, 84112, United States

**Keywords:** Adolescent, Child, Exercise, Occupational groups, Schools, Technology

## Abstract

**Objective:**

School students and office workers can have higher levels of physical inactivity. Comparative effectiveness of interventions that use technology-based strategies to promote physical activity (PA) between these two populations is lacking. The purpose of this review was to compare effectiveness of technology-based interventions to promote PA in school and office settings.

**Methods:**

A literature search was conducted from each database's inception with no place restriction using PubMed/MEDLINE (1946), Scopus (2004), Web of Science (1964), Embase (1974), and Cochrane Library (1995) through January 1st, 2025. Inclusion criteria included randomized controlled trials examining school-aged children/adolescents or adult office workers participating in a technology-based intervention to promote PA. Effectiveness was examined using random-effects meta-analyses.

**Results:**

Ten school-based and five office-based studies were examined. Two of 10 (20%) school-based studies and two of five (40%) office-based studies showed high risk of bias. There was a significant but small positive effect for school-based interventions to improve PA (Hedges' g = 0.35, 95%CI [0.02, 0.68]) but not for office-based interventions (Hedges' g = 0.07, 95%CI [−0.19, 0.33]).

**Conclusions:**

School-based interventions using technology showed a small positive effect for improving PA. More novel, undisruptive, and effective strategies using technology should be derived and rigorously tested in school and office settings.

## Highlights

• School interventions using technologies are effective to promote physical activity.
• There is large heterogeneity in school interventions using technology.
• Office-based studies using technology does not show effectiveness.
• There are limited quality physical activity interventions using technology.
• More physical activity interventions using technology need testing.

## 1 Introduction

Physical activity (PA) has numerous benefits for children, adolescents, and adults, including a healthy cardiometabolic profile, higher levels of cognitive functioning, and good mental and emotional health (Organization WH, 2025; Lee, 2009; DER and SSD, 2017). The World Health Organization recommends that children and adolescents average at least 60 min of PA per day throughout the week with three days of muscle and bone strengthening activities (Bull et al., 2020). Less than 20% of youth meet these recommendations (Guthold et al., 2020). Schools serve as a logical focal point for PA intervention, with youth spending almost 20% of their weekly time in school (approximately 30–35 h per week) (Hall and Nielsen, 2020). Schools thus have the potential to exert a large influence on behavior (Lounsbery et al., 2013). This is especially important for PA behavior change because “a lack of time” is considered one of the largest barriers to adhere to PA recommendations as most of the school day is spent being inactive (Bragg et al., 2009; Johnson et al., 2023). Unfortunately, a recent systematic review found that youth typically spend about two-thirds of their school day sedentary, especially during academic class time, between classes, during lunch, and being transported to and from school (Egan et al., 2019).

Office workers are similarly constrained to long periods of inactivity (Smith et al., 2015; Parry and Straker, 2013). There are deleterious effects on health from low PA including higher risk of all-cause mortality, in addition to a higher incidence of cancer, cardiovascular disease, and type II diabetes (Biswas et al., 2015). Yet, akin to school-aged youth, office workers also claim a “lack of time” as the largest barrier to engaging in PA (Abdul Manaf et al., 2021). By failing to comply with the guidelines of at least 150 min/week of moderate-to-vigorous physical activity (MVPA) and at least two days/week of muscle strengthening activities (WHO Guidelines Approved by the Guidelines Review Committee, 2020), office workers miss out on PA's physical, cognitive, and emotional benefits (Sjøgaard et al., 2016).

To facilitate the health of communities, it is imperative that effective interventions to promote PA are utilized in places where individuals learn and work. Both school-aged youth and adult office workers have structured days that comprise a large amount of inactivity. Although there are contextual differences between these populations, use of technology has the potential to promote PA (Amini et al., 2015; Flodgren et al., 2020; Militello et al., 2018; Abdin et al., 2018; Chu et al., 2016; Parés-Salomón et al., 2024), however, no study to date has been untaken to quantify effectiveness of PA interventions using technology to promote PA in these two distinct settings. In schools, there exists a breadth of intervention types to improve PA including classroom activity breaks to provide additional PA opportunities throughout the school day, enhancement of lunchtime and recess programs, and physical education curriculum changes (Naylor et al., 2015). In office settings, appointments with health professionals, individual or team-based goal-setting, educational materials, pedal desks, reinforcement emails, mobile phone applications, and wearable technology are popular modalities. While adherence to these technology features is high, there may not necessarily be sizeable effects on PA behavior change as many interventions use technology for assessment purposes only or to reduce sedentary behavior. It is important for reviews on this topic to focus on active engagement for PA promotion. PA has effects on health distinct from sedentary behaviors like sitting, thus a review must be undertaken to examine the effects of technology specifically on increasing PA (not decreasing sedentary behavior) in school and office settings. Therefore, the purpose of this study is to systematically review the literature and compare the effectiveness of technology-based PA interventions delivered at school and office settings to improve PA in primary and secondary students and office workers, respectively.

## 2 Methods

### 2.1 Search strategy

This systematic review with meta-analysis was conducted in alignment with the Preferred Reporting Items for Systematic Reviews and Meta-Analyses (PRISMA) guidelines. The review protocol was registered in PROSPERO (CRD420251048769). A literature search was conducted from each database's inception using PubMed/MEDLINE (1946), Scopus (2004), Web of Science (1964), Embase (1974), and Cochrane Library (1995) through January 1st, 2025. There were no place restrictions. We used the following keywords though Truncation and Boolean logic searching. For school-based interventions, the search words (“schools” OR “physical education”) AND (“physical activity” OR “exercise”) AND (“child” OR “adolescent” OR “student”) AND (“technology” OR “ehealth” OR “mhealth”) AND (“controlled trials”) were used. For office-based interventions, the search words (“physical activity” OR “exercise”) AND (“office worker” OR “desk worker” OR “sedentary worker” OR “workplace”) AND (“technology” OR “ehealth” OR “mhealth”) AND (“controlled trials”) were used. Only English language-based journals were considered. Two authors (M.B. and R.D.B.) screened and extracted relevant studies independently. Any disagreements were resolved through discussion until consensus.

### 2.2 Inclusion criteria

We used the “Population Intervention Comparison/Control Outcome Study Design” framework for the basis of our inclusion criteria. Studies were included in this review if they met all of the following criteria: Population: school-aged children or adolescents (6–17 years old) and adult office workers; Intervention: school-based or office-based PA interventions using technology for PA promotion during school or work hours with no restrictions on the duration, frequency, or type of PA; Comparison: comparison groups that did not receive any additional PA interventions; Outcome: PA assessed using either self-report or device-based methods; Study design: randomized controlled trials (RCTs) only. We chose 6 years old as the lower bound age limiter because primary school (elementary) typically starts at age 6, which is the first grade in the US. If a study was modified during COVID-19 to deliver a technology-based intervention and this change was clearly indicated in the title and abstract of a screened study, that study would be included in the analysis if all other criteria were met.

### 2.3 Study data extraction

Two authors independently extracted relevant data from eligible studies that included: study characteristics, participant characteristics, including age, sex, and school grade; intervention details, including setting, type, intensity, session duration, frequency, total duration, and follow-up duration; type of technology used to promote PA; measurements and outcomes of PA.

### 2.4 Quality assessment

The risk of bias for each study was assessed using the Revised Cochrane Collaboration's Revised Tool to Assess Risk of Bias in Randomized Trials (RoB2) (JAC et al., 2019). Each domain was assessed as low risk, some concerns, or high risk of bias for each extracted study. An overall rating was also given for each study. Assessed domains were weighted the same and consisted of bias arising from randomization, timing and recruitment of the participants, deviation from the intended intervention, missing data, measurement of outcome data, and selection of reported results. High risk of bias indicated a high potential of low or poor experimental validity due to the use of weaker methods related to each of the assessed domains.

### 2.5 Statistical analysis

Study summaries and results from the risk-of bias assessment were communicated descriptively as counts and percents (%s) within the main text. Quantitative data from each study were organized to analyze group level sample sizes, group mean differences, and standard deviations of the mean differences. To examine effectiveness, a random effects meta-analysis was employed that accounted for both between study heterogeneity and within-study sampling error. Specifically, we used the Sidik-Jonkman random effects model because of its accurate performance in meta-analyses with large heterogeneity (Sidik and Jonkman, 2007). We also used the Hartung-Knapp-Sidik-Jonkman error adjustment because of the use of meta-analyses with only a few studies (IntHout et al., 2014). Meta-analyses were conducted for the total sample (i.e., school- and office-based combined) and for each setting separately. Comparisons between school-based and office-based interventions was tested using meta-regression. Individual study effects and the overall pooled effect were reported using a Forest Plot using the bias-corrected effect size measure (Hedges'g) to quantify the effects. Effect sizes were considered small if g < 0.2, medium if g = 0.05, and large if g > 0.80. Heterogeneity across the studies was assessed using Cochran's Q test and the I^2^ statistic (Higgins et al., 2003). The magnitude of between-study heterogeneity was determined small if I^2^ < 50%, moderate if I^2^ = 50%–75%, and large if I^2^ > 75%. Publication bias was assessed using Funnel Plots, the Egger linear regression test, and a Galbraith plot (Egger et al., 1997). A post hoc sensitivity analysis was conducted, removing a single study from the meta-analysis per iteration to determine if the standardized mean differences would significantly change. Alpha level was set at *p* < 0.05 and all analyses were conducted using Stata v.19.0 statistical software package (Statacorp., College Station, TX, USA).

## 3 Results

### 3.1 Study characteristics

**Fig. 1:**
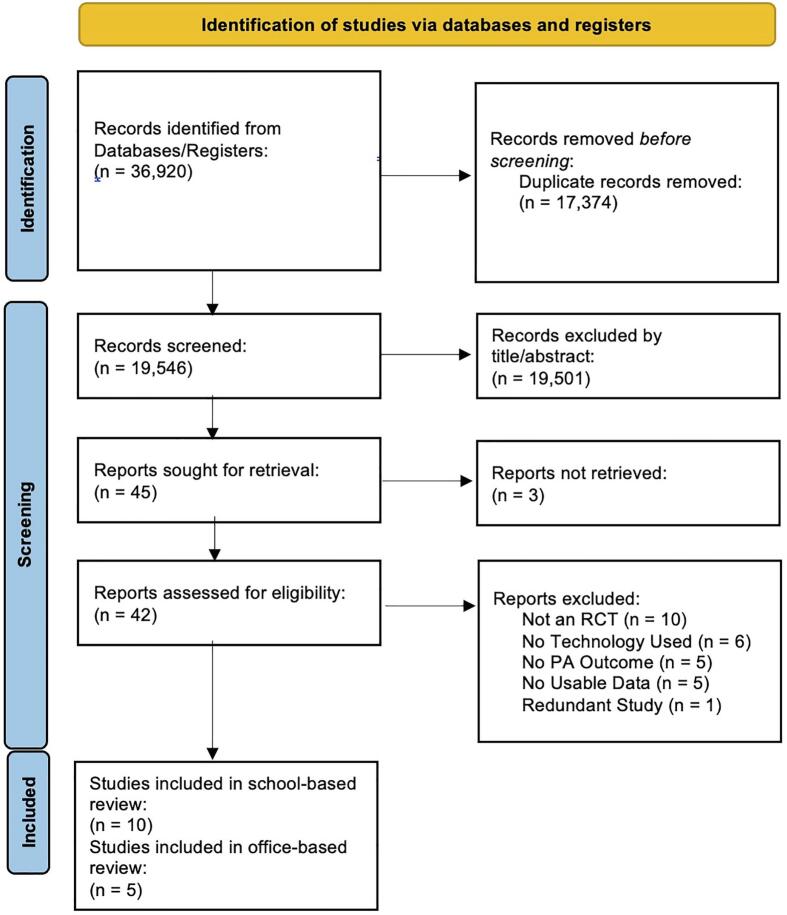
PRISMA flow diagram for extracting technology enhanced school- and office-based physical activity intervention studies identified using PubMed/MEDLINE, Scopus, Web of Science, Embase, and Cochrane Library from database inception through January 1, 2025. *Note:* The literature search was conducted from each database's inception using PubMed/MEDLINE (1946), Scopus (2004), Web of Science (1964), Embase (1974), and Cochrane Library (1995) through January 1st, 2025.

The results of the systematic search are presented in Fig. 1 and study summaries are reported within Table 1. We identified and analyzed 10 school-based and five office-based studies.^S1-S15^ Within the school-based studies, three were conducted in Australia,^S1,S2,S5^ four in Europe,^S3,S4,S7,S8^ and three in the US·^S6,S9,S10^ All 10 school-based studies were clustered RCTs.^S1-S10^ Intervention durations ranged from a single 30 min session to three years. Participants' average age was 13.4 years old. Out of the 10 studies, four studies used self-report as the assessment of PA,^S3,S4,S9,S10^ four studies reported device-based PA outcomes,^S2,S5-S7^ and two studies reported a combination of self-report and device-based PA outcomes.^S1,S3^ Within the office-based studies, one study was conducted in Australia,^S15^ two in Europe,^S11,S14^ one study from China,^S12^ and one in the US·^S13^ Of the 5 studies,^S1-S5^ three used clustered RCTs,^S11,S12,S14^ and two studies used traditional RCTs.^S13,S15^ Intervention durations ranged from eight weeks to six months. Participants' average age was 44.0 years old. Four studies reported device-based assessments of PA,^S11,S13-S15^ and one used self-report.^S12^

**Table 1:** Study summary table of extracted technology enhanced school- and office-based physical activity intervention studies identified using PubMed/MEDLINE, Scopus, Web of Science, Embase, and Cochrane Library from database inception through January 1, 2025.

Author	Country	Sample Characteristics	Behavior Change Technique	Technology Used	Intervention Group	Control Group	Outcome(s)	Key Findings
School-based Studies								
Caillaud et al. (2022)	Australia	2 schools *N* = 83 Age range = 10–12 years old Intervention *n* = 57 Control *n* = 26	Goal setting, self-monitoring, performance feedback; No theory stated	Tablet with iEngage app: activity tracker and interactive learning modules.	10 digital modules were delivered focusing on experiential learning, goal setting and self-assessments	No PA program.	MVPA and daily steps	Intervention showed 30% increase in daily steps.
Dewar et al. (2013)	Australia	12 schools *N* = 357 girls Mean age = 13.2 ± 0.5 years Intervention *n* = 179 Control *n* = 178	Education, self-monitoring; positive reinforcement; Social Cognitive Theory	Text messages.	Enhanced school sport sessions; interactive seminars; lunch-time PA; handbooks and pedometers; parent newsletters; text messaging	No PA program.	Accelerometer counts/min; % of time in MVPA.	Not significant.
Ezandam et al. (2012)	Netherlands	20 schools *N* = 883 Age range = 12–13 years Intervention *n* = 485 Control *n* = 398	Education, self-assessment, personalized feedback; Multi-theoretical	Web-based computer-tailored intervention.	Eight computer modules for behavior change.	Regular curriculum	Self-reported PA.	Positive effect on step counts at 2-year follow-up.
Haerens et al. (2007)	Belgium	15 schools *N* = 1194 Mean age = 13.1 ± 0.8 years old Intervention *n* = 911 Control *n* = 735	Extending school PA opportunity time, parental involvement; No theory stated	Computer-tailored feedback.	Changed physical environment; Sports materials; Computer-tailored intervention; **Parent support.**	No PA program.	Self-reported PA.	The intervention with parental support led to an increase in only self-reported school-related PA.
Lubans et al. (2009)	Australia	6 schools N total = 124 Mean age = 14.1 years old Intervention *n* = 58 Control *n* = 66	Education, self-monitoring, personalized feedback; Social Cognitive Theory	Pedometers and emails.	10-week school sports program; information sessions on PA; pedometer monitoring; PA handbooks and monthly parent emails.	10-week sports program.	Step counts.	Increased step counts.
Prochaska et al. (2004)	United States	One school N total = 138 Mean age = 12.1 years old Intervention *n* = 46 Control *n* = 46	Education, computerized assessment, personalized feedback; No theory stated	Computer program.	Computerized health assessment followed by tailored feedback.	No PA program.	Device measured PA.	PA showed significant improvement in Boys.
Tymms et al. (2015)	England	60 schools *N* = 1494 Age range = 11–12 years old Intervention *n* = 340 Control *n* = 393	Peer mentoring, education, self-assessment, personalized feedback; No theory stated	GIS, accelerometer, and GPS units.	6 weekly meetings with students. IG2–6 weekly geography lessons.	No PA program.	Device-measured PA.	Higher PA in participative learning condition.
Van Woudenberg et al. (2018)	Netherlands	One School Mean age = 12.2 *N* = 190 Intervention *n* = 93 Control *n* = 97	Influencing Agents, peer modeling, education; Self Determination Theory	Smartphone, MyMovez application and Fitbit Flex.	Most influential peers were nominated and trained to promote PA via smart phones.	No PA program.	Device-measured PA.	Steps decreased in both groups from baseline to post-intervention.
Velicer et al. (2013)	United States	20 schools Age range = 11–14 years old *N* = 4158 Intervention *n* = 2184 Control *n* = 1974.	Education, personalized feedback; Transtheoretical Model of Behavior Change	Computer	Five 30-min computerized TTM-tailored sessions.	Substance prevention program.	Self-reported PA frequency.	Greater number of days of PA.
Whittamore et al. (2012)	United States	3 schools Mean age = 15.3 *N* = 384 Intervention *n* = 207 Control *n* = 177	Education, goal setting, self-monitoring, health coaching, social networking; Social Learning Theory	Computerized lessons and blogs.	8 lessons with individualized feedback of self-assessment, set goals, interactive health.	No PA program.	Self-reported PA.	Increased PA over 6 months.
Office-based Studies								
Aiattasalo et al. (2012)	Southern Finland	20 worksites Mean age = 44.1 years old *N* = 241 Intervention *n* = 118 Control *n* = 123	Self-monitoring, education; Health Action Process Approach	*E*-mails.	One group meeting; log-monitored pedometer-use; six e-mail messages.	No PA program.	Walking behaviors.	Proportion of walkers significantly increased.
Blake et al. (2019)	China	2 Information Technology Organizations Age range 35–40 years old *N* = 690 Intervention *n* = 490 Control *n* = 200	Prompts, exercise demonstrations, peer modeling, education; No theory stated.	Website demonstrating Qigong exercises.	Six exercise videos posted every 2-weeks on website.	No PA program.	Self-report PA (IPAQ).	No significant differences between groups.
Gell et al. (2015)	United States	One public university Mean age = 48.9 ± 10.6 years old *N* = 87 Intervention n = 46 Control n = 41	Education, self-regulation; self-efficacy promotion Behavior Choice Theory	Text messages.	3 motivational text messages per week for 24 weeks.	No PA program.	Steps/day	No significant difference at 24-weeks.
Hunter et al. (2018)	Ireland	9 public sector organizations Mean age = 43.0 ± 10.0 years old *N* = 853 Intervention *n* = 457 Control *n* = 396	Goal setting, self-monitoring, personalized feedback, positive reinforcement; Leaning Theory	Websites, WIFI beacons (sensors) and PAL key fob.	WIFI beacons to encourage PA.	No PA program.	Step counts	Mean steps/day were significantly lower in the intervention group.
Neuhaus et al. (2014)	Australia	One university Age range = 20–65 years old *N* = 44 Intervention *n* = 30 Control n = 14	Message Prompts, environment modification, encouragement; Social Cognitive Theory	Telephone support.	Height-adjustable workstations; management consultation; staff education; manager e-mails to staff; **individual-level***coaching*; *telephone support.*	No PA program.	Stepping time, Device-measured PA.	No significance.

*Note:* The literature search was conducted from each database's inception with no place restriction using PubMed/MEDLINE (1946), Scopus (2004), Web of Science (1964), Embase (1974), and Cochrane Library (1995) through January 1st, 2025. PA = physical activity, MVPA = moderate-to-vigorous physical activity, IPAQ = International Physical Activity Questionnaire, WIFI=Wireless Fidelity, GIS = Geographic Information System, PAL = physical activity leader.

### 3.2 Types of technology and coverage

One school-based study used tablets,^S1^ two used smart phones,^S2,S8^ and six used computers.^S3-S6,S9,S10^ One study used Geographical Information Systems (GIS) technology, which is a computer-based framework for capturing, storing, analyzing, managing, and displaying all types of geographically referenced data.^S7^ For office-based interventions, two used email messaging,^S11,S15^ one used beacon sensors used around the workplace,^S14^ one used websites,^S12^ and one used text messaging.^S13^

Overall, the technologies used across studies can be grouped into four functional categories: (1) feedback-based technologies, (2) communication-based technologies, (3) immersive/spatial technologies, and (4) educational computer-tailored modules. Across both school- and office-based settings, technology was primarily used to deliver information, provide personalized feedback, and support motivation, with fewer studies employing immersive or spatially oriented technologies. Specifically, feedback-based technologies were used in several school-based studies that used computers to deliver theory-based modules providing information about the link between behavior and health while utilizing personalized feedback to sustain motivation.^S3,S4,S9,S10^ Communication-based technologies were used in school-based studies used text messaging to provide PA information with motivational messaging,^S2^ to communicate PA promotion strategies to influencing agents (e.g., peer “team captains”),^S8^ and in three office-based studies that used e-mail messaging to provide PA information and promote PA in the workplace.^S11,S13, S15^ For immersive technologies, one school-based study used GIS in students' classrooms and neighborhoods to help them learn about their spatial movement patterns, how the environment shapes behaviors, and to use the spatial data to set personal goals,^S7^ and an office-based study used workplace beacon sensors to record PA data to assess workers' behaviors and to use the data for personalized goal setting.^S14^ Finally, educational computer-tailored modules were used in a school-based study that used tablets with an app that communicated PA-related concepts with a link to activity trackers to facilitate the connection between PA concepts and experiences,^S1^ and in an office-based study used websites to deliver modules to workers regarding performing Qigong exercises.^S12^

### 3.3 Quantitative results

Risk of bias from school-based and office-based studies is shown in Supplemental Files (Fig. S1 and Fig. S2). Within the school-based studies, six (60%) were assessed to have low risk of bias,^S4-S6, S8-S10^ two (20%) had some concerns,^S1,S3^ and two (20%) had high risk of bias.^S2,S7^ For office-based studies, one (20%) was assessed as low risk of bias,^S14^ two (40%) assessed as some concerns,^S11,S12^ and two (40%) assessed as high risk of bias.^S13,S15^

**Fig. 2:**
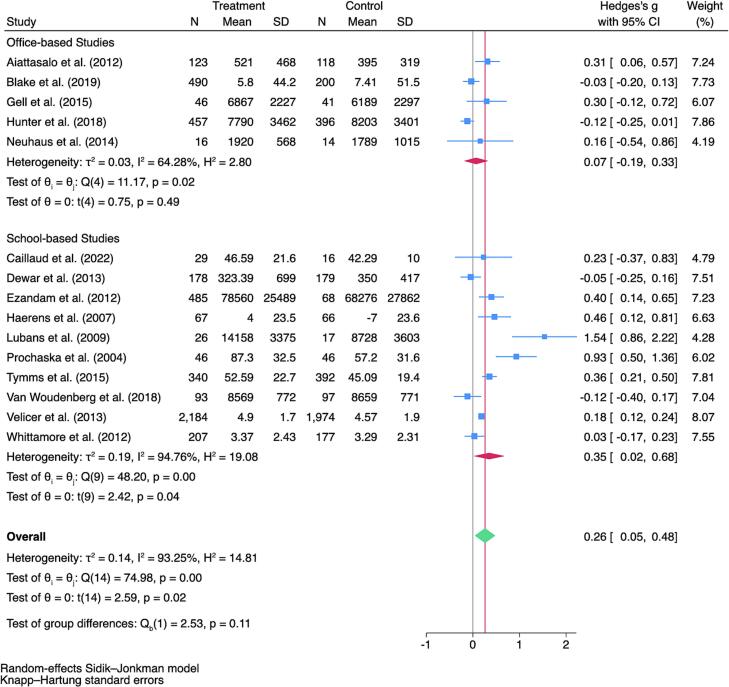
Forest plot of technology enhanced school- and office-based physical activity intervention studies identified using PubMed/MEDLINE, Scopus, Web of Science, Embase, and Cochrane Library from database inception through January 1, 2025. *Note:* The literature search was conducted from each database's inception using PubMed/MEDLINE (1946), Scopus (2004), Web of Science (1964), Embase (1974), and Cochrane Library (1995) through January 1st, 2025.

The pooled effectiveness of the interventions was shown within a Forest Plot in Fig. 2. Results showed a small but significant positive effect overall (Hedges' g = 0.26, 95%CI [0.05, 0.48]), primarily driven by school-based studies (Hedges' g = 0.35, 95%CI [0.02, 0.68]). Office-based interventions' effect was not significant (Hedges' g = 0.07, 95% CI [−0.19, 0.33]). Moderate to large heterogeneity was observed (Total I^2^ = 93.3%; School-based I^2^ = 94.8%; Office-based I^2^ = 64.3%). These results suggest that technology used in school settings for the promotion of PA is statistically different from 0 but small in magnitude. For office-based studies, use of technology did not influence the promotion of PA.

**Fig. 3:**
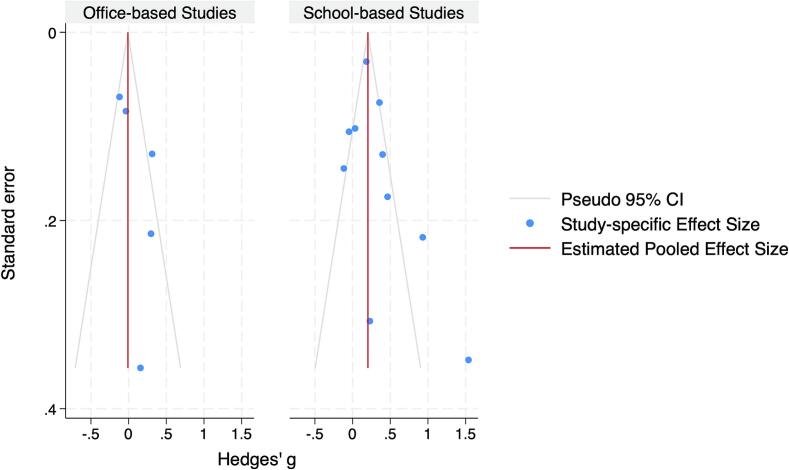
Funnel plots of examined technology enhanced school- and office-based physical activity intervention studies identified using PubMed/MEDLINE, Scopus, Web of Science, Embase, and Cochrane Library from database inception through January 1, 2025. *Note:* The literature search was conducted from each database's inception using PubMed/MEDLINE (1946), Scopus (2004), Web of Science (1964), Embase (1974), and Cochrane Library (1995) through January 1st, 2025.

Egger's regression test showed significance for small-study effects using the total sample (β₁ = 2.33, *p* = 0.03). The office-based interventions showed no evidence of publication bias (Fig. 3), Egger's test was non-significant (β₁ = 1.5, *p* = 0.20), and the Galbraith plot (Supplemental Fig. S3) revealed no outliers or strong trends. The separate analysis for the school-based interventions revealed some evidence of small-study effects (β₁ = 3.10, *p* = 0.03) with a slightly asymmetrical Funnel Plot (Fig. 3) and a Galbraith Plot showing a linear trend (Supplemental Fig. S3).

The effect of intervention setting (school vs. office) showed non-significance (β = 0.23, 95% [−0.20, 0.66]). These results indicated that there were no differences on the effect of technology for promoting PA in school and office settings.

Finally, we performed a leave-one-out sensitivity analysis with results presented within the Supplementary File (Supplemental Fig. S4). Leaving any one study out did not change the significance for the overall pooled effect (all *p*'s < 0.05), or the office-based studies (all *p*'s > 0.05). For the school-based studies, leaving out Prochaska et al.^S6^ lowered the pooled effect to becoming marginally significant (Hedges' g = 0.29, 95%CI [0.00, 0.58]).

## 4 Discussion

The present study systematically reviewed the literature and compared the effectiveness of technology-based PA interventions used for PA promotion in schoolchildren and office workers. The salient finding from this review was that a positive but small, statistically significant effect was observed within the pooled analysis, which was primarily driven by school-based interventions; however, there was significant level of heterogeneity across the reviewed studies. Prior reviews examined the effectiveness of eHealth interventions for promoting PA across settings and found no significant pooled effects on PA (Sequí-Domínguez et al., 2024). The authors concluded that despite the rapid recent developments in technology, its positive impact on PA behaviors lacks robust evidence. Champion et al. (Champion et al., 2019) conducted another review examining the effects of multi-behavioral eHealth interventions specifically in school settings and observed small but significant pooled effects for PA. However, the risk of bias for many of the studies were high with the authors concluding weak evidence to support the utility of technology to promote PA during school.

Regarding office-based studies, no significant pooled effects were observed. A systematic review examining PA interventions in shift workers found no studies examined behavioral changes as an outcome (Flahr et al., 2018), precluding any inferences to be made regarding PA interventions' effect on behavior change. Indeed, the current review observed only one of the five extracted studies reporting positive significant effects for technology to improve PA.^S11^ Paradoxically, one office-based intervention even observed a significant inverse effect on step counts within the experimental group.^S14^ Evidence to suggest effects on PA improvement are small with perhaps some evidence for the utility of email prompts to promote walking.^S11^ Unlike a review conducted by Pares-Salomon et al. (Parés-Salomón et al., 2024) who found that multicomponent interventions using digital features are effective in decreasing sedentary time, our study did not identify any significant effect specifically on increasing PA. Similarly, a review conducted by Jung & Cho suggested that mHealth interventions are effective for improving PA among all workers (Jung and Cho, 2022). Again, this contrasts with our work, but we specifically examined office-based workers. The lack of positive effect for increasing PA in office workers may be related to the design of the interventions to only reduce sedentary time. Different methods mat be needed to promote PA rather than just decrease sedentary behavior in office workers.

Six out of 10 school-based studies communicated significant positive effects; however, the studies showed significant heterogeneity, which makes the utility of the observed findings questionable. School-based interventions tended to focus more on the promotion of PA and not just focus on the reduction of sedentary behavior. The studies that showed the strongest effects in schools tended to be grounded in an established theoretical framework. Four out of the five office-based studies communicated a theoretical framework, and six of the 10 school-based studies used a theoretical framework for intervention development with common frameworks being Self -Determination Theory and Social Cognitive Theory (Ryan and Deci, 2000; Bandura, 2004). Effective interventions used computers (via emails and websites) to communicate information and prompts, provide support for behavior change, and self-assessment. Longer interventions that utilized an exposure to the intervention over several months tended to yield stronger effects in addition to interventions that incorporated parental social support. The strongest effect was observed by Lubans et al.^S5^ in the *Program X* intervention. The intervention was conducted over six months, using pedometers as a measurement tool for physical activity. Bandura's social cognitive theory served as the theoretical framework for this intervention and included five main components: (1) enhanced school sports programs, (2) information sessions, (3) pedometers, (4) PA and nutrition handbooks and monthly informational newsletters for parents, and (5) social support for healthy behavior via email.^S5^ Prochaska et al.^S6^ provided the second strongest effect among included studies. Participants were students among classrooms in one middle school that completed a computerized health assessment, with two experimental conditions. In one condition, feedback was provided only for PA behavior, and in the other condition, feedback was provided for diet as well as PA. The intervention was limited to a one-time exposure lasting about 30 min and the follow up period was four months. This study separated cohorts by gender and only found significant intervention effects for boys in both conditions. These results suggest that among school-aged children and adolescents, tailoring interventions by gender may be necessary. RoB was evaluated as low risk. Haerens et al.^S4^ also found significant effects of their nine-month long intervention in both boys and girls when the intervention was combined with parental support. Computer tailoring in this intervention was used to individualize active lifestyle promotion for students. Environmental changes were also implemented among all schools that included increased access to PA opportunities and extra sports materials, including additional sports equipment. For five out of 10 included schools, parental involvement was included to create a supportive social environment. The parents completed a computer-tailored PA activity at home in this group. Investigators found that the intervention condition plus parental support resulted in higher school related PA. RoB2 was evaluated as low risk.

Ezandam et al.^S3^ utilized a web-based intervention including eight modules with information regarding balance-related behavior, personalized feedback, prompts for intention formation, as well as instructions and suggestions for change. These 15-min modules were delivered over 10 weeks, with follow-up periods of four months and two years. Multiple theories were incorporated, and an overall positive effect size was observed at the two-year follow-up. Authors stated that results surrounding PA from this intervention are inconclusive, and PA promotion may require additional environmental changes. Tymms et al.^S7^ tested two separate interventional designs, delivered over six weeks: peer mentoring without technology, and participative learning with a technology-based component. GIS was used to allow students in this group to collect and interpret data about their own physical activity and consider how their environment was impactful. A small but significant effect size was observed for participants in the technology-based intervention. The participative learning group increased their MVPA compared to control. No significant effect was observed for the peer mentoring group. While the more modest PA intervention without a technology component showed no significant effect, allowing student autonomy in interpreting their PA behavior through the GIS intervention appeared to have a positive effect. RoB2 was evaluated to be high risk, with concerns for missing outcome data, timing, and selection of reported results.

Finally, Velicer et al.^S9^ studied the longest intervention from baseline to closeout, with sessions being delivered over three years from 6th to 8th grade. Participants filled out annual assessments and were given transtheoretical model tailored feedback at each time point. The PA outcome measure was self-reported number of days per week which they received at least 60-mintues of PA. At 36-months, the intervention group reported a higher number of PA days compared to control. This study provided evidence that computer-tailored interventions are effective at influencing PA behavior, despite logistical challenges in delivery at the time of intervention.

Moderation analysis yielded no significant prediction of effect size when testing for setting differences. Other sources of heterogeneity may have been PA outcome measurement type, duration of the intervention, dose and frequency of the implemented technology, type of technology used, and differences between sexes and among age groups. Given these results, it is unclear if these two populations respond differently to the use of technology to promote PA. We need more interventions employed in office settings that are designed specifically to increase PA. Time constraints during the workday and perhaps a higher degree of autonomy during the school day for students may also be factors why school-based studies showed some higher level of effectiveness for promoting PA relative to office-based studies. But again, because of the lack of statistical differences between settings using meta-regression, possibly due to the small sample sizes, it is uncertain if these potential factors have an impact.

Based on the results of this review, several recommendations can be provided for intervention development. School-based interventionalists should consider tailoring their programs by gender to improve outcomes for girls, who are more likely to be non-responders. For office-based studies, there may have been differences in compliance to intervention protocols, differences arising due to scheduling, worker motivation, demographic differences, and office worker type. Given that social support leads to higher PA, deriving interventions with a strong social support component to supplement technology use is recommended in office settings. Additionally, office-based interventions may benefit from gender specific programming given that gender-specific tailoring of interventions tends to elicit stronger effects. Intervention developmentalists should consider the effects of duration on outcomes and long-term retention of behavior change since habit formation typically takes at least several weeks (Singh et al., 2024). More modernized technology, such as Artificial Intelligence (abbreviated “AI”), provides the opportunity for exploration of intervention methodologies in both settings. AI chatbots for PA behavior change are in its early stages of development, but its communication style can facilitate its use as a health coach or a self-monitoring support tool.

Limitations to this review included examining only school- and office-based interventions and not PA interventions targeting the out-of-school or after-work settings. The school-based studies showed a high level of heterogeneity, which limits the interpretability of the pooled effect sizes from the meta-analyses. Interventions may have been implemented differently with varying levels of fidelity. Furthermore, many school-based studies used self-report methods to assess PA, which may have lowered the construct validity of the assessments. Finally, there were a limited number of recently published studies using RCTs. Most of the screened studies were observational or pilot studies, which did not reach the criteria for quantitative analysis. There may have been non-RCT's that were not included in this review that may have provided relevant information regarding effectiveness of these types of PA interventions.

This review has several notable strengths including the review and comparison of PA interventions specifically to promote PA in two distinct settings that tend to facilitate lower levels of PA throughout the day. Our specific inclusion criteria made us able to make precise conclusions regarding the quality and effectiveness of these interventions relative to other reviews on this topic. Robust meta-analytical approaches were employed to obtain valid effect size estimates, and a thorough risk of bias assessment was performed to characterize study quality.

## 5 Conclusion

Overall, there were a small number of published experimental studies that used technology to promote PA in school and office settings. Of the studies that were extracted, school-based PA interventions that used technology showed a small positive pooled effect on PA behavior; however, no pooled effect was observed for office-based studies. Effective school-based interventions used computers for education and self-assessment, social support to facilitate behavior change, and longer length interventions to facilitate habit formation. Again, these results should be interpreted with caution as there was high heterogeneity among school-based studies. It is recommended that more novel and effective strategies using technology be derived and rigorously tested in school and office settings to promote PA.

## CRediT authorship contribution statement

**Mitch Wyatt:** Writing – original draft, Methodology, Investigation, Conceptualization. **Mickey Bolyard:** Writing – original draft, Methodology, Investigation, Conceptualization. **Lingyi Fu:** Writing – original draft, Software, Methodology, Formal analysis, Conceptualization. **Hayley Almes:** Writing – original draft, Investigation, Conceptualization. **Frank Adutwum:** Writing – original draft, Investigation, Conceptualization. **Charles Rodgers:** Writing – original draft, Investigation, Conceptualization. **Ryan D. Burns:** Writing – original draft, Supervision, Methodology, Investigation, Formal analysis, Conceptualization.

## Funding

This research did not receive any specific grant from funding agencies in the public, commercial, or not-for-profit sectors. All authors report no real or perceived conflicts of interest related to this study.

## Declaration of competing interest

The authors declare that they have no known competing financial interests or personal relationships that could have appeared to influence the work reported in this paper.

## Data Availability

Data will be made available on request.

## Appendix A Supplementary data

Supplementary material Image 1
