# Supplementary material for: Comparative effectiveness of school- and office-based technology-enhanced interventions for physical activity promotion: A systematic review and meta-analysis
Source: Prev Med Rep. 2026 Feb 13;63:103409. doi: 10.1016/j.pmedr.2026.103409 (PMC12924117; doi:10.1016/j.pmedr.2026.103409)
Supplement: Supplementary file 1 — Supplementary material [file mmc1.docx]

**Table S1.** References of reviewed studies.

| School-based Studies | S1. Caillaud C, Ledger S, Diaz C, et al. iEngage: A digital health education program designed to enhance physical activity in young adolescents. *PLoS One*. 2022;17(10):e0274644. doi:10.1371/journal.pone.0274644 |
| --- | --- |
|  | S2. Dewar DL, Morgan PJ, Plotnikoff RC, et al. The nutrition and enjoyable activity for teen girls study: a cluster randomized controlled trial. *Am J Prev Med*. 2013;45(3):313-317. doi:10.1016/j.amepre.2013.04.014 |
|  | S3. Ezendam NP, Brug J, Oenema A. Evaluation of the Web-based computer-tailored FATaintPHAT intervention to promote energy balance among adolescents: results from a school cluster randomized trial. *Arch Pediatr Adolesc Med*. 2012;166(3):248-255. doi:10.1001/archpediatrics.2011.204 |
|  | S4. Haerens L, De Bourdeaudhuij I, Maes L, et al. School-based randomized controlled trial of a physical activity intervention among adolescents. *J Adolesc Health*. 2007;40(3):258-265. doi:10.1016/j.jadohealth.2006.09.028 |
|  | S5. Lubans DR, Morgan PJ, Callister R, Collins CE. Effects of integrating pedometers, parental materials, and E-mail support within an extracurricular school sport intervention. *J Adolesc Health*. 2009;44(2):176-183. doi:10.1016/j.jadohealth.2008.06.020 |
|  | S6. Prochaska JJ, Sallis JF. A randomized controlled trial of single versus multiple health behavior change: promoting physical activity and nutrition among adolescents. *Health Psychol*. 2004;23(3):314-318. doi:10.1037/0278-6133.23.3.314 |
|  | S7. Tymms PB, Curtis SE, Routen AC, et al. Clustered randomised controlled trial of two education interventions designed to increase physical activity and well-being of secondary school students: the MOVE Project. *BMJ Open*. 2016;6(1):e009318. doi:10.1136/bmjopen-2015-009318 |
|  | S8. Van Woudenberg TJ, Bevelander KE, Burk WJ, et al. Testing a social network intervention using vlogs to promote physical activity among adolescents: a randomized controlled trial. *Front Psychol*. 2020;10:2913. doi:10.3389/fpsyg.2019.02913 |
|  | S9. Velicer WF, Redding CA, Paiva AL, et al. Multiple behavior interventions to prevent substance abuse and increase energy balance behaviors in middle school students. *Transl Behav Med*. 2013;3(1):82-93. doi:10.1007/s13142-013-0197-0 |
|  | S10. Whittemore R, Jeon S, Grey M. An internet obesity prevention program for adolescents. *J Adolesc Health*. 2013;52(4):439-447. doi:10.1016/j.jadohealth.2012.07.014 |
| Office-based Studies | S11. Aittasalo M, Rinne M, Pasanen M, et al. Promoting walking among office employees - evaluation of a randomized controlled intervention with pedometers and e-mail messages. *BMC Public Health*. 2012;12:403. doi:10.1186/1471-2458-12-403 |
|  | S12. Blake H, Lai B, Coman E, et al. Move-It: a cluster-randomised digital worksite exercise intervention in China: outcome and process evaluation. *Int J Environ Res Public Health*. 2019;16(18):3451. doi:10.3390/ijerph16183451 |
|  | S13. Gell NM, Wadsworth DD. The sue of text messaging to promote physical activity in working women: a randomized controlled trial. *J Phys Act Health*. 2015;12(6):756-763. doi:10.1123/jpah.2013-0144 |
|  | S14. Hunter RF, Gough A, Murray JM, et al. *A loyalty scheme to encourage physical activity in office workers: a cluster RCT*. Southampton (UK): NIHR Journals Library; 2019. |
|  | S15. Neuhaus M, Healy GN, Dunstan DW, et al. Workplace sitting and height-adjustable workstations: a randomized controlled trial. *Am J Prev Med*. 2014;46(1):30-40. doi:10.1016/j.amepre.2013.09.009 |


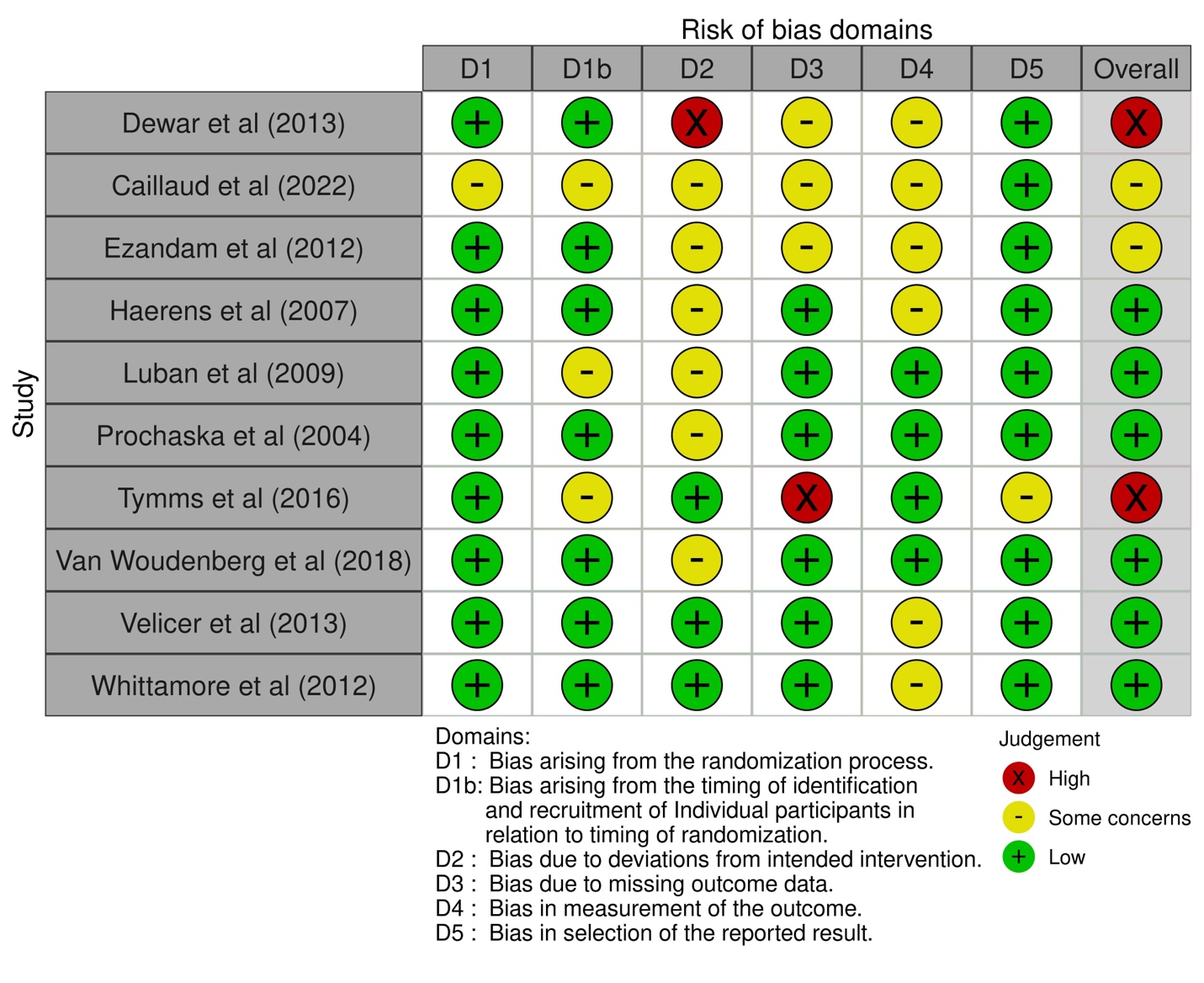


**Supplemental Figure S1.** Risk-of-bias assessment for school-based studies from a search through January 1st, 2025.


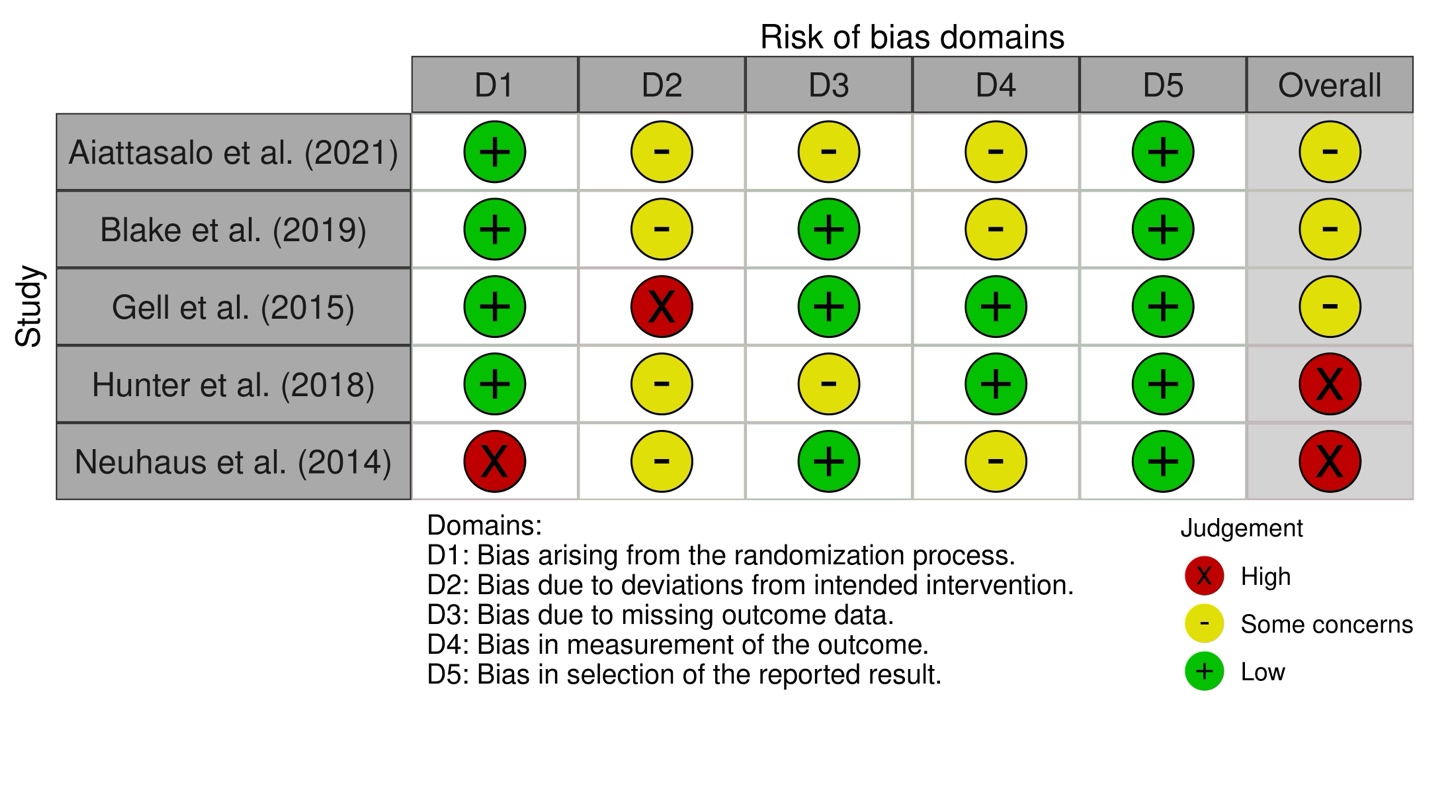


**Supplemental Figure S2.** Risk-of-bias assessment for office-based studies from a search through January 1st, 2025.


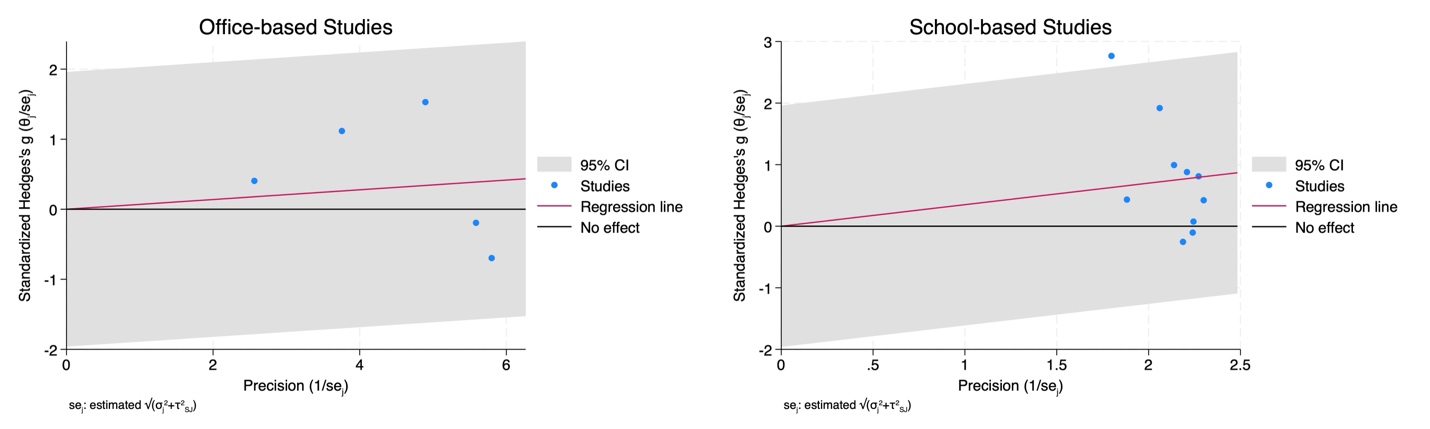


**Supplemental Figure S3.** Galbraith plots for school- and office-based studies from a search through January 1^st^, 2025.


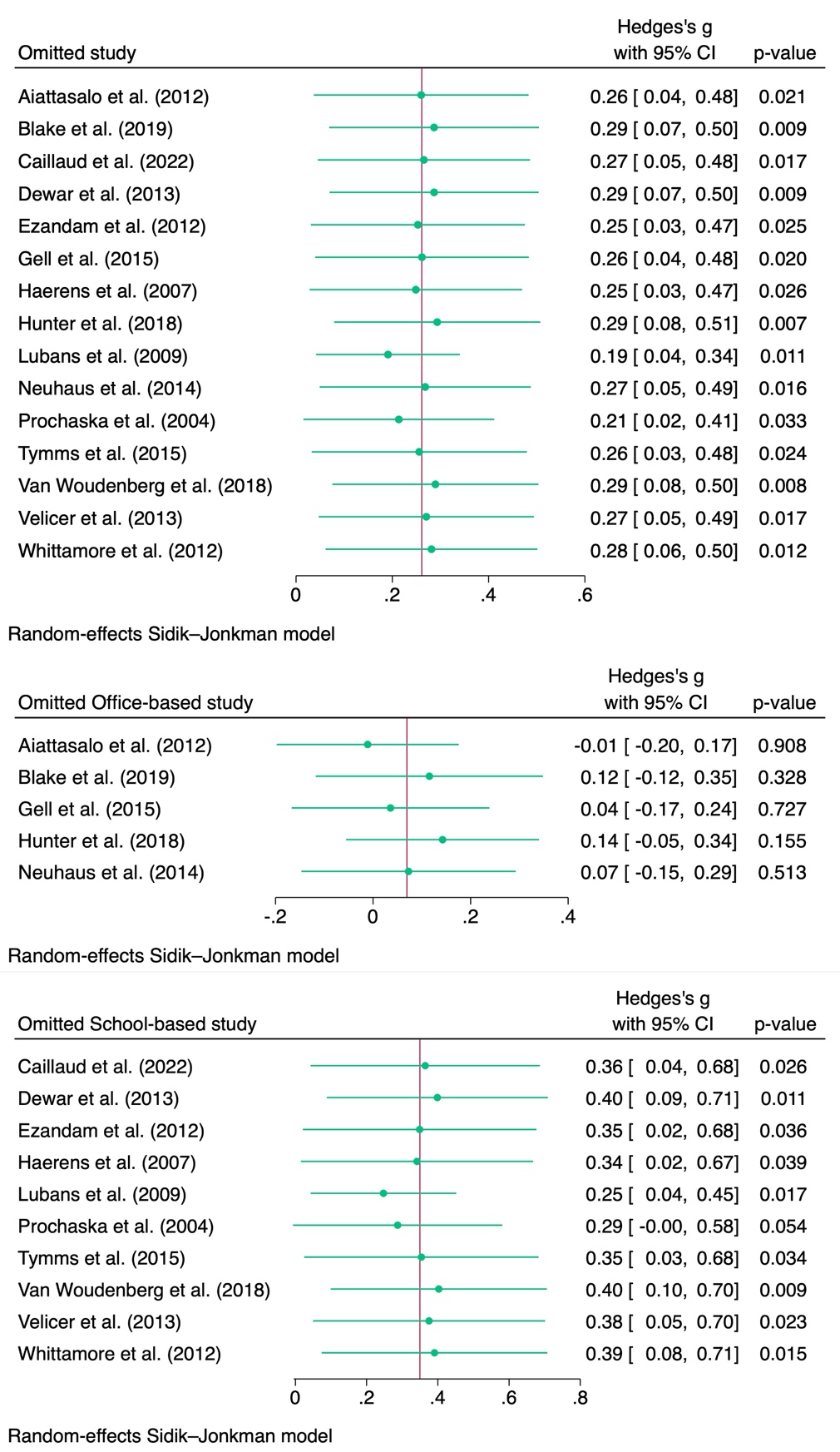


**Supplemental Figure S4.** Change in pooled effects the total sample (top panel), office-based studies (middle panel), and school-based studies (bottom panel) using leave-one-out methodology.
